# The economic impacts of social activism and corporate social responsibility on food fraud

**DOI:** 10.1371/journal.pone.0304153

**Published:** 2024-06-11

**Authors:** Anubrata Deka, Amalia Yiannaka, Konstantinos Giannakas

**Affiliations:** 1 Department of Economics, Flame University, Pune, Maharashtra, India; 2 Department of Agricultural Economics, University of Nebraska-Lincoln, Lincoln, Nebraska, United States of America; ’Enrico Fermi’ Research Center, ITALY

## Abstract

The study examines the relationship between the corporate social responsibility (CSR) investments of a food firm, an activist’s incentive to target the firm to uncover and deter fraudulent behavior, and the firm’s incentive to commit food fraud. Specifically, we develop a game theoretic model to analyze the strategic interaction between a food firm that decides whether to provide a credence food attribute and whether to misrepresent the quality of its product, and an activist who decides whether to monitor the firm and launch a campaign to uncover and remove false/misleading quality claims. We further examine the effect of CSR and the activist’s presence on the level of quality the firm provides. We derive the conditions under which an activist will find it optimal to monitor the firm to uncover fraudulent quality claims and the firm will find it optimal to misrepresent its product quality. Analytical results show that the greater the firm’s CSR investments, the less likely it is that the activist will find it optimal to monitor the firm, and the more likely it is that the firm will find it optimal to misrepresent its product quality. Results also show that the firm is more likely to misrepresent its product quality when its effectiveness in contesting the activist’s campaign is relatively high, and more likely to actually provide a high-quality product when the cost of the credence attribute is relatively low.

## 1. Introduction

Food firms’ engagement in corporate social responsibility (CSR) has been increasing in recent years, taking multiple forms which range from reducing plastic waste to tackling youth unemployment. (In 2011 only 20% of the companies on the S&P 500 index published a CSR report, whereas 90% of companies on the S&P 500 index published a CSR report in 2019 [1, 2].) A firm’s CSR investments are voluntary/proactive, can be motivated by moral duty or self-interest [3], and can be a response to exogenous factors such as governmental regulations and/or pressure from different activist organizations [4–7]. Engagement in proactive CSR can enhance a firm’s relationship with the public by building its reputation of being a responsible citizen, trustworthy and credible. Studies have argued that through CSR activities, firms can develop a reputation that provides ‘insurance-like’ protection from future negative publicity and attacks on the firm [8, 9]. However, firms engaging in CSR practices may choose to exploit this public trust by engaging in fraudulent behavior.

Food fraud which includes food adulteration (e.g., ingredient substitution, dilution or concealment) and mislabeling (e.g., misrepresentation of the quality and type of the food product) poses a serious threat to the integrity of the global food industry [10, 11]. (The percentage of product fraud that occurs globally is estimated at 5–7% of world trade, resulting in annual costs of $40 billion [12]. See Giannakas and Yiannaka [11] for a discussion of the causes and consequences of food fraud, strategies actors along the supply chain employ to combat food fraud, and a comprehensive review of recent food fraud incidents.) Mislabeling, the most common type of food fraud [13, 14], has received increasing activist attention in recent years, including from legal activism. Citing lack of regulatory oversight and the need to protect consumers from deceptive food labels and marketing practices, activist groups have been playing an increasingly active role in the filing of lawsuits against food and beverage firms [15, 16]. Interestingly, many of the contested claims involve use of socially responsible practices such as ‘humane and environmentally responsible production’ and providing ‘a safe work environment’ [15].

Indeed, many food firms that have invested in CSR have been under scrutiny by different activist organizations that accuse these firms of making false and misleading claims about their products and services. For instance, Nestlé has invested heavily in CSR activities, ranging from reducing plastic waste to educating pregnant women and young children on the importance of health and nutrition. (In India, Nestlé supported the project Jagriti, collaborated with a local NGO named Mamta Health Institute of Mother and Child, with the primary objective of creating peer mentor groups that educate and counsel pregnant and lactating mothers on the importance of healthy nutrition, initiation of exclusive breastfeeding, and improved breastfeeding practices.) At the same time, however, Nestlé has been accused by two activist organizations, the Changing Markets Foundation (CMF) and the International Baby Food Action Network (IBFAN), for making false claims about its infant formula product being a close substitute for breastmilk and for breaking established standards set by the World Health Organization (WHO) and UNICEF [17].

The potential effect of a food firm’s investment in CSR on its incentives to engage in fraudulent behavior has not been previously examined. Relatedly, the effect of CSR on the activists’ incentives to target a firm when their objective is to expose fraudulent behavior and increase market transparency is not obvious. On the one hand, a firm’s CSR investment may make it harder for activist organizations to gain support from the public when they launch a campaign against the firm (e.g., a boycott) due to the goodwill the firm has created through its CSR activities [8]. This can decrease the activists’ incentive to monitor and target the firm and may increase the firm’s incentive to engage in fraudulent behavior. On the other hand, King and McDonnell [18] find that simply belonging to the top tier of the most reputable firms attracts unwanted activist attention and makes it more likely to be targeted by an activist. This is primarily because, when activists target firms with strong reputations, they could attract more public attention to their cause. This is especially true for food firms because the food sector fulfills basic human needs and is essential to human survival and safety. Food consumers are highly opinionated with respect to the production processes of, and the products provided by the food sector [19]. In this context, firms that have invested in CSR may expect more scrutiny by activists, which may decrease their incentive to engage in fraudulent behavior.

The purpose of this study is to shed light on the above issues by formally modeling and analyzing the relationship between a food firm’s CSR investments, its decision to engage in food fraud, and an activist’s decision to target the firm to expose and deter fraudulent behavior. A few studies have examined the factors that influence an activist’s target selection, and the role CSR may play in affecting an activist’s decision to target a firm. Baron [3] examined the type of firms the activist will target when firms differ in their motivation to invest in CSR. The study finds that if citizens cannot determine a firm’s true motivation for providing CSR, an activist is more likely to target a firm that is morally motivated. Baron [20] further analyzed the target selection of different types of activists, where targets differ in their degree of vulnerability. The study finds that a moderate activist is more likely to target more vulnerable firms whereas a radical activist is more likely to target relatively less vulnerable firms. The study also finds that by self-regulating, firms can reduce the likelihood of being targeted in the future. King and McDonnell [18] analyzed whether reputation building and increasing visibility by adopting prosocial activities affect the likelihood of being targeted by an activist. The study finds that belonging to the top-tier of the most reputable firms attracts unwanted activist attention, making it more likely to be targeted by an activist.

The present study extends the literature by analyzing the effect of CSR on a food firm’s incentive to commit food fraud, and an activist’s decision to target the food firm to expose and deter fraudulent behavior. While Baron [20] and King and McDonnell [18] assume that the activist chooses her target with the objective of improving the firm’s voluntary provision of CSR, our study examines how CSR affects the activist’s incentive to monitor and challenge the firm when the activist’s objective is to uncover and remove fraudulent quality claims. The study further examines the effect of CSR and the activist’s presence on the quality claims the firm makes and on the level of quality it provides. Specifically, the study develops a game theoretic model of strategic interaction between a firm that decides whether to provide a credence quality attribute and the claims it will make about the quality it provides, and an activist who decides whether to monitor the firm and, if she detects fraud, whether to launch a campaign to remove false and/or misleading quality claims. The study derives the conditions under which the activist will find it optimal to monitor the firm to uncover false quality claims and the firm will find it optimal to misrepresent its product quality. The rest of the study is structured as follows: section 2 presents the theoretical model, section 3 provides the analytical solutions and section 4 discusses model extensions and concludes the study.

## 2. The model

We consider a market where two food products are produced, good *h* which may offer a credence quality attribute, and good *k* which is a basic good that does not offer the quality attribute. There are two firms in the market, firm *H* that produces good *h* and firm *K* that produces good *k*, a continuum of consumers who differ in the strength of their preference for the credence quality attribute, and an activist.

Firm *H* chooses the quality of good *h* it will produce, that is, whether to provide the credence attribute or not, denoted by *s*_*a*_ = 1 and *s*_*a*_ = 0, respectively. (An example of this decision could be whether to use a specific production process such as organic production, sustainable production practices, or green energy sources.) Provision of the quality attribute is costly, and we assume these costs are fixed and given by *F*_*h*_ > 0. Firm *H* also decides what claims it will make about the quality it produces. We denote the quality claimed by *s*_*c*_ ≥ *s*_*a*_ where *s*_*a*_ is the actual quality level produced by firm *H*. Thus, if the firm claims *s*_*c*_ > *s*_*a*_ it engages in fraudulent behavior, that is, the firm claims to provide the credence attribute when it does not produce it, which implies that *s*_*a*_ = 0 and *s*_*c*_ = 1. If the firm claims *s*_*c*_ = *s*_*a*_ it does not commit fraud as it claims to provide the quality that it actually produces; in this case *s*_*c*_ is either *s*_*c*_ = *s*_*a*_ = 1 or *s*_*c*_ = *s*_*a*_ = 0. Moreover, we assume that variable production costs are zero and both firms choose their prices to maximize profits. We also assume that firm *H* has invested in CSR which is denoted by $Z\in R^{+}$ and these investments have occurred prior to the firm choosing the quality of good *h*.

We further assume that there is a continuum of heterogeneous consumers who prefer the credence attribute that could be provided by firm *H*. The differentiating consumer attribute reflects the strength of preference for the credence attribute, it is denoted by *λ*, and it is assumed to be uniformly distributed with unit density *f*(*λ*) = 1 in the interval *λ* ∈ [0,1]. In this market, each consumer buys one unit of either good *h* or good *k* and their purchase represents a small share of their budget. Given the above, consumer utility can be represented as:

$$U_{h}=U-p_{h}+{\;s}_{c}\lambda$$ (1)

$$U_{k}=U-p_{k}$$ (2)

where *U* is a base level of utility associated with the consumption of the two products, *p*_*h*_ and *p*_*k*_ is the price of good *h* and *k*, respectively, and *s*_*c*_ is the quality of the credence attribute claimed by firm *H* as defined previously. We assume that the base level of utility is such that the market is served by at least one product, i.e., *U* ≥ *p*_*i*_ where *i* ∈ (*h*,*k*).

The objective of the activist is to increase market transparency by uncovering market fraud and removing false and/or misleading quality claims. The utility the activist obtains when she succeeds in removing fraudulent quality claims is denoted by B > 0, and the utility she obtains when, after monitoring, she finds that firm *H* has truthfully claimed its quality (i.e., there is no fraud) is denoted by *β* > 0, where B > *β*. We thus assume that the activist benefits more when she can remove fraudulent quality claims because success in removing fraud can boost her reputation as an effective activist, leading to greater trust and donations from the public, which enable her to fund and launch future campaigns. The utility the activist obtains when the campaign fails is given by *L*, where *β* > *L*. Increasing market transparency is costly for the activist and these costs include monitoring costs, denoted by *c*_*m*_, and the cost/effort of launching a campaign against the firm, denoted by *e*.

To uncover fraud and remove fraudulent quality claims, the activist will first choose whether to monitor firm *H*. We assume that if firm *H* claims that it does not provide the credence attribute (i.e., *s*_*c*_ = 0), then the activist does not have a reason to monitor and will not monitor the firm. When firm *H* claims that it provides the credence attribute (i.e., *s*_*c*_ = 1), the activist decides whether to monitor the firm or not anticipating that there is a likelihood, denoted by *μ*, that the firm’s claims are fraudulent where 0 < *μ* ≤ 1. If, during monitoring, the activist detects fraud (i.e., a discrepancy between the actual and the claimed quality, *s*_*c*_ > *s*_*a*_), then she directs social pressure on firm *H* to change the claims it makes about its quality. The social pressure exerted by the activist takes the form of the activist launching a campaign against the firm wherein she will choose the level of effort to exert, *e*. (Public support in a campaign launched by an activist can take the form of boycotting a firm’s product, donating to the activist’s campaign and/or organization, taking part in protests against the firm, and signing petitions initiated by the activist.) A campaign is successful when the activist convinces consumers that the firm has committed food fraud, which results in the firm no longer being able to make fraudulent quality claims. (Among the numerous examples of firms making fraudulent quality claims (see Giannakas and Yiannaka [11] for examples of recent mislabeling incidents), some receive widespread public attention and have costly reputation repair consequences for the offending firms, such as the Volkswagen emissions scandal.) We thus assume that prior to the uncovering of the fraudulent behavior by the activist, consumers have no reason to doubt firm *H*’s quality claims; we discuss the implications of relaxing this assumption later in the paper.

The campaign launched by the activist represents a threat to firm *H* primarily because it increases its cost through the effort it has to exert, denoted by *f*, to counter the activist’s claims, and reduces its profitability when the campaign succeeds and the firm has to truthfully claim a lower product quality. If the campaign fails, which implies that the activist was unsuccessful in convincing consumers that the firm engaged in fraudulent behavior, firm *H* maintains its pre-campaign claimed quality of the credence attribute. We assume that, regardless of whether the activist’s campaign succeeds or not, the firm does not face other types of penalties by regulators; we discuss the implications of relaxing this assumption later in the paper. It is important to note that firms may not face penalties for misrepresenting the quality of their credence attributes when well-defined standards regarding the provision of the credence attributes do not exist (e.g., claims that the product is sustainably or ethically produced). In fact, this may also be the case for attributes whose provision involves clearly defined standards (e.g., organic production). (A case in point involves an investigation by the Washington Post of Aurora Organic Dairy, one of the largest organic milk suppliers in the US, which claimed that the dairy supplier violated organic standards and sold conventional milk as organic to the largest grocery chains [10, 11, 21]. The US Department of Agriculture (USDA) conducted an investigation and, despite finding that annual audits by the certifier were conducted after grazing season, violating USDA inspection rules, allowed the firm to keep its USDA organic label concluding that the firm’s *current* livestock and pasture management practices complied with USDA organic regulations [22].)

We further assume that firm *H*’s CSR investments increase the effectiveness of the effort exerted by the firm to contest a campaign against it because the involvement of the firm with CSR activities creates goodwill and leads to greater public support making it harder for the activist to convince the public that the firm has engaged in fraudulent behavior. This assumption is in line with the findings of Godfrey et al. [8] who find that CSR investments can provide insurance like protection to firms against negative publicity and future attacks. In our model, this is captured by *α*(*Z*)*f*, where *α*(*Z*) > 1 is the effectiveness of the firm in contesting the campaign and it is positively related to the CSR investments of the firm, that is, *α*′(*Z*) > 0. The functional form of *α*(*Z*) will determine the extent to which CSR affects the firm’s effort to contest the activist’s campaign, which in turn depends on the idiosyncrasies of the firm, the activist, and the food sector they operate in. As an example, when *α*(*Z*) is given by the simple functional form *α*(*Z*) = *δ* + *Z*, where *δ* ≥ 1, the firm’s CSR investments improve its effectiveness in contesting the activist’s campaign at a constant rate.

The outcome of the campaign is uncertain, and it is determined by the Nash equilibrium of a contest game played between the activist and firm *H* as in Baron [23]. The outcome of the contest game is determined by a contest success function, which is dependent on the level of effort exerted by both parties and the effectiveness of the effort exerted by the firm relative to the activist. Specifically, the probability of success of the campaign is denoted by *θ* and it increases in the effort exerted by the activist (*e*) and decreases in the effort exerted by the firm (*f*) and in the firm’s effectiveness in contesting the campaign *α*(*Z*), such that $\theta =\frac{e}{e+\alpha (Z)f}$.

Table 1 summarizes the key parameters of the model and, where applicable, describes the relationship between them.

**Table 1:** Key model parameters.

Notations	Meanings	Range of values	Parameter relationships
*s*_*a*_	The quality of the credence attribute firm *H* actually provides	*s*_*a*_ = {0,1}	
*s*_*c*_	The quality of the credence attribute firm *H* claims to provide	*s*_*c*_ = {0,1}	*s*_*c*_ ≥ *s*_*a*_
*F*_*h*_	Fixed cost of providing the credence attribute	*F*_*h*_ > 0	
*μ*	Likelihood that firm *H*’s claims are fraudulent	0 < *μ* ≤ 1	
*c*_*m*_	Activist’s cost of monitoring the firm	*c*_*m*_ ≥ 0	
*e*	Effort the activist exerts during the campaign	*e* ≥ 0	
*f*	Effort firm *H* exerts during the campaign	*f* ≥ 0	
*Z*	Firm *H*’s CSR investments	*Z* > 0	
*α*(*Z*)	The effectiveness of firm *H*’s effort in contesting the campaign	*α*(*Z*) > 1	*α*′(*Z*) > 0
*B*	Activist utility when the campaign succeeds	*B* ≥ 0	
*β*	Activist utility when after monitoring she finds that firm *H* has truthfully claimed its quality	*β* ≥ 0	*β* < *B*
*L*	Activist utility when the campaign fails	*L* ≥ 0	*L* < *β* < *B*
*θ*	Probability of campaign success	0 ≤ *θ* ≤ 1	$\theta =\frac{e}{e+\alpha (Z)f}$

**Fig 1:**
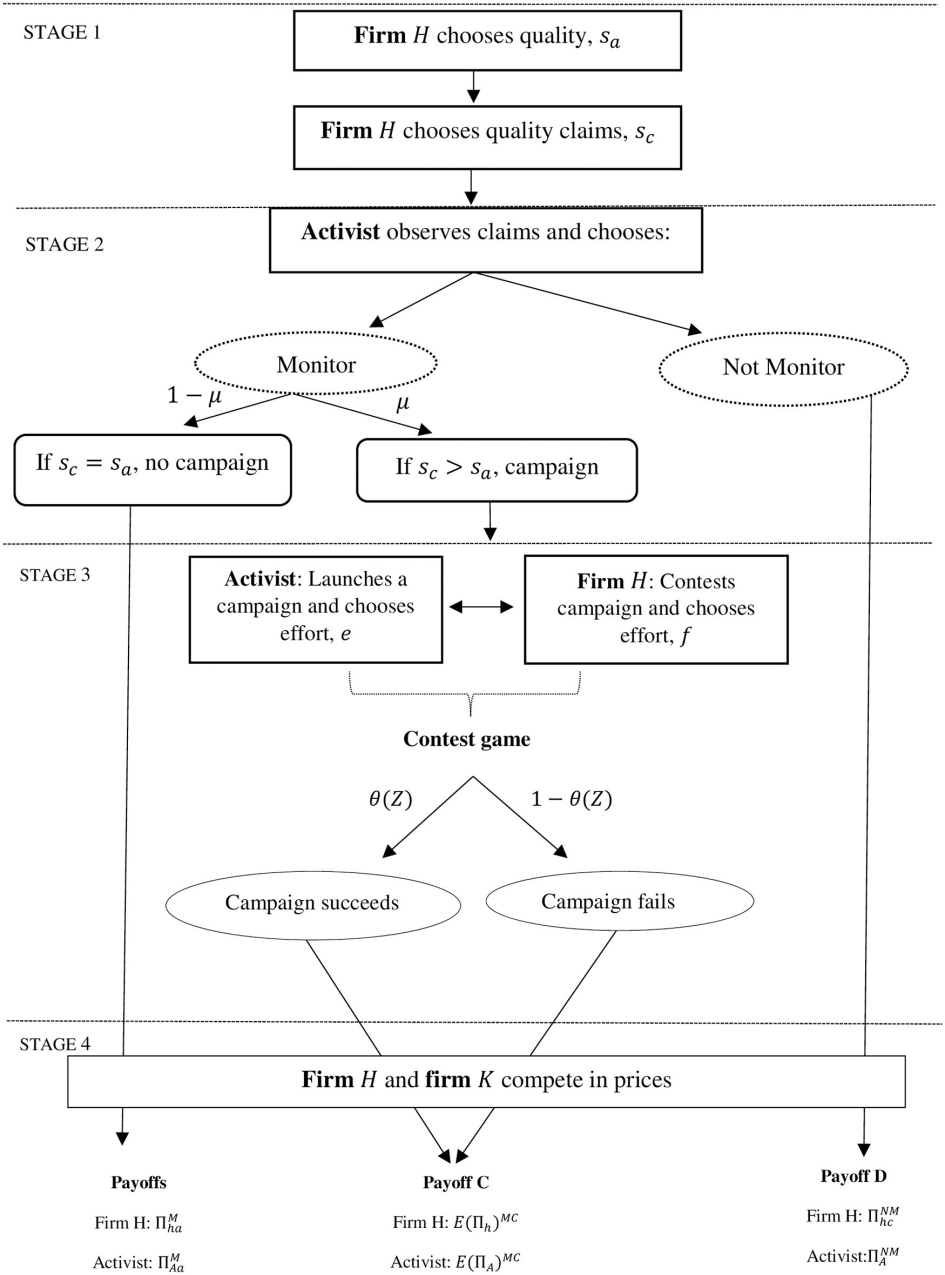
The game tree of the interaction between firm *H* and the activist.

The strategic interaction between firm *H* and the activist is modeled as a four-stage game and is depicted in Fig 1. In the first stage, firm *H* first chooses the quality of the good it will produce and then chooses the claims it will make about the product’s quality. In the second stage, after observing the quality claimed by the firm, the activist chooses whether to monitor the firm or not. As discussed previously, if firm *H* has not claimed to provide the credence quality attribute, *s*_*c*_ = 0, the activist does not monitor the firm since there is no fraud to be investigated and there is no further interaction between the activist and firm *H*. If the activist observes that firm *H* has claimed to provide the credence quality attribute, *s*_*c*_ = 1, the activist decides whether to monitor the firm given that there is likelihood *μ* that the firm’s claims are fraudulent. If the activist chooses not to monitor the firm, firms compete in prices and the game ends. If the activist chooses to monitor the firm, she will conduct costly research to compare the claimed quality to the actual quality of good *h*. The activist launches a campaign against the firm if the claimed quality is greater than the actual quality, i.e., *s*_*c*_ = 1 and *s*_*a*_ = 0, otherwise, no campaign is launched, the firms compete in prices and the game ends. If the activist launches a campaign against the firm, then, in the third stage, a contest game is played between the activist and the firm where each player chooses their level of effort. There are two potential outcomes of the contest game, that is, the campaign launched by the activist can either succeed with probability *θ* or it can fail with probability 1 − *θ*. If the campaign succeeds, firm *H* will change its quality claims and truthfully claim its actual quality, while if the campaign fails, firm *H* will continue making the same quality claims. Finally, after the contest outcome has been realized, firms choose their prices and consumers choose the products they will consume.

## 3. Analytical results

The game is solved using backwards induction to derive the sub-game perfect equilibrium.

### Stage 4—The pricing decisions

In the last stage, firms compete in prices and consumers choose the products they will consume. The following outcomes are possible depending on whether the activist monitors the firm and, if so, whether a campaign is launched; the market equilibrium is determined for each of these possible outcomes.

- The activist does not monitor firm *H*

If firm *H* claims to provide the credence attribute, *s*_*c*_ = 1, and the activist does not monitor the firm, firm *H* does not have to change its quality claims. Consumer utility under this situation is given by Eqs (1) and (2) where *U*_*h*_ = *U*_*hc*_ is the consumer utility from the consumption of good *h*, *U*_*k*_ = *U*_*kc*_ is the consumer utility from the consumption of the basic good *k*, and *p*_*h*_ = *p*_*hc*_ and *p*_*k*_ = *p*_*kc*_ are the prices of good *h* and good *k*, respectively. The consumer with differentiating attribute *λ*_*c*_: *U*_*hc*_ = *U*_*kc*_ ⇒ *λ*_*c*_ = *p*_*hc*_ − *p*_*kc*_ is indifferent between good *h* and good *k*, while consumers with *λ* ∈ [0,*λ*_*c*_) values prefer good *k* and consumers with *λ* ∈ (*λ*_*c*_,1] values prefer good *h*. Assuming that the indifferent consumer consumes good *k*, the demand for good *h* and *k* is given by *x*_*hc*_ = 1 − *λ*_*c*_ = 1 − (*p*_*hc*_ − *p*_*kc*_) and *x*_*kc*_ = *λ*_*c*_ = *p*_*hc*_ − *p*_*kc*_, respectively.

Both firms choose prices with the objective of maximizing profits as:

$$\begin{array}{l} \underset{p_{hc}}{\text{max}\pi_{hc}}=p_{hc}\left(1-\left(p_{hc}-p_{kc}\right)\right)\;\\ \underset{p_{kc}}{\text{max}\pi_{kc}}=p_{kc}\left(p_{hc}-p_{kc}\right) \end{array}$$ (3)

The Nash equilibrium prices and the resulting quantities and profits are given by:

$$\begin{array}{l} Firm\;H:\;p_{hc}^{*}=\frac{2}{3},\;x_{hc}^{*}=\frac{2}{3},\;\pi_{hc}^{*}=\frac{4}{9}\\ Firm\;K:\;p_{kc}^{*}=\frac{1}{3},\;x_{kc}^{*}=\frac{1}{3}\;,\;\pi_{kc}^{*}=\frac{1}{9} \end{array}$$ (4)

The payoff of the activist is given by $\Pi_{A}^{NM}=0$.

Recall that the activist will not monitor firm *H* when it does not claim to provide the credence quality attribute, *s*_*c*_ = 0. If firm *H* claims *s*_*c*_ = 0, neither firm provides the credence attribute and, given Bertrand competition between the two firms, the firms share the market ($x_{hc}^{*}=x_{kc}^{*}=\frac{1}{2}$) and earn zero profits ($p_{hc}^{*}=p_{kc}^{*}=0$ and $\pi_{hc}^{*}=\pi_{kc}^{*}=0$). The activist would not realize any benefits under this outcome. When this outcome is compared to the outcome in Eq (4), it is easy to see that the profits of both firms are increasing in the quality claims made by firm *H*.

- The activist monitors and does not launch a campaign

The activist does not launch a campaign if monitoring reveals that firm *H* has not made fraudulent quality claims, i.e., when *s*_*c*_ = *s*_*a*_. Both firms choose prices with the objective of maximizing profits, where *π*_*ha*_ and *π*_*ka*_ are the profits of firm *H* and firm *K*, respectively. In this case, the Nash equilibrium prices, and the resulting quantities and profits are identical to those derived in Eq (4), where the activist does not monitor and firm *H* claims to provide the credence quality attribute. The payoff of the activist that monitors firm *H* but does not launch a campaign is given by:

$$\Pi_{Aa}^{M}=\beta -c_{m}$$ (5)

- The activist monitors and launches a campaign

If, after monitoring, the activist finds that firm *H* has made false claims, that is *s*_*c*_ > *s*_*a*_, then the activist launches a campaign against firm *H*, which can succeed with probability *θ* or fail with probability (1 − *θ*).

#### The campaign succeeds

If the campaign succeeds (i.e., the activist is successful in convincing the public that the firm has engaged in fraudulent behavior), firm *H* must change its quality claims and claim its actual quality. Both firms choose prices to maximize their profits, where *π*_*hs*_ and *π*_*ks*_ are the profits of firm *H* and firm *K*, respectively, when the campaign succeeds and, because both firms provide the same product, they earn zero profits. The payoff of the activist when the campaign succeeds is given by:

$$\pi_{As}^{MC}=Β-c_{m}-e$$ (6)

#### The campaign fails

If the campaign fails (i.e., the activist is unsuccessful in convincing the public that the firm has engaged in fraudulent behavior), firm *H* will not change its claims about the quality it produces. Both firms choose prices to maximize profits, where *π*_*hf*_ and *π*_*kf*_ are the profits of firm *H* and firm *K*, respectively, when the campaign fails. In this case, the Nash equilibrium prices, and the resulting quantities and profits are identical to those derived in Eq (4), where the activist does not monitor and firm *H* claims to provide the credence quality attribute. The payoff of the activist when the campaign fails is given by:

$$\pi_{Af}^{MC}=L-c_{m}-e$$ (7)

### Stage 3—A campaign is launched against firm *H*

At this stage of the game the activist launches a campaign where she chooses the level of effort, *e*, she will exert to apply pressure on firm *H* to change its quality claims. At the same time firm *H* chooses the level of effort, *f*, that it will exert to contest the campaign against it. The outcome of the campaign is determined by the level of effort exerted by both parties and the effectiveness of the effort exerted by the firm relative to the activist, *α*(*Z*). Specifically, the probability of success of the campaign is given by:

$$\theta =\frac{e}{e+\alpha (Z)f}$$ (8)

where all variables are as defined previously. Note that, if the firm’s CSR investments had no impact on the firm’s effort in contesting the campaign (i.e., *α*(*Z*) = 1), then the probability of campaign success would be given by $\theta =\frac{e}{e+f}$, which implies that the activist and the firm are equally effective during the campaign.

#### Contest game

The activist chooses the level of effort to maximize the expected payoff given by:

$$\begin{array}{cc} \underset{e}{\text{max}}\;E{\left(\Pi_{A}\right)}^{MC} & =\theta \pi_{As}^{MC}+\left(1-\theta \right)\pi_{Af}^{MC}=\left(\frac{e}{e+\alpha \left(Z\right)f}\right)\pi_{As}^{MC}+\left(\frac{\alpha \left(Z\right)f}{e+\alpha \left(Z\right)f}\right)\pi_{Af}^{MC}\\ & =\left(\frac{e}{e+\alpha \left(Z\right)f}\right)Β+\left(\frac{\alpha \left(Z\right)f}{e+\alpha \left(Z\right)f}\right)L-c_{m}-e \end{array}$$ (9)

Similarly, firm *H* will choose the level of effort to maximize its expected profits given by:

$$\begin{array}{cc} \underset{f}{\text{max}}\;{E(\Pi_{h})}^{MC} & =\theta \pi_{hs}+\left(1-\theta \right)\pi_{hf}-f=\left(\frac{e}{e+\alpha \left(Z\right)f}\right)\pi_{hs}+\left(\frac{\alpha \left(Z\right)f}{e+\alpha \left(Z\right)f}\right)\pi_{hf}-f\\ & =\left(\frac{e}{e+\alpha \left(Z\right)f}\right)\;0+\;\left(\frac{\alpha \left(Z\right)f}{e+\alpha \left(Z\right)f}\right)\left(\frac{4}{9}\right)-f \end{array}$$ (10)

The first order conditions (FOC) of the optimization problems are:

$$\begin{array}{l} \frac{\alpha \left(Z\right)f}{{\left(e+\alpha \left(Z\right)f\right)}^{2}}\left[B-L\right]=1\\ \frac{e}{{\left(e+\alpha \left(Z\right)f\right)}^{2}}\left(\frac{4}{9}\right)=1 \end{array}$$ (11)

Solving the FOC simultaneously gives the optimal level of effort for firm *H* and the activist as:

$$f^{*}=\frac{(B-L)\alpha (Z){\left(\frac{4}{9}\right)}^{2}}{{\left[(B-L)+\alpha (Z)\left(\frac{4}{9}\right)\right]}^{2}}$$ (12)

$$e^{*}=\frac{{\left((B-L)\right)}^{2}\alpha (Z)\left(\frac{4}{9}\right)}{{\left[(B-L)+\alpha (Z)\left(\frac{4}{9}\right)\right]}^{2}}$$ (13)

It is straightforward to show that the optimal effort the activist exerts during the campaign, e*, increases in the benefits the activist could realize when the campaign succeeds, *B*, and decreases in the benefits realized when the campaign fails, *L*. The optimal effort of the firm and the activist can increase or decrease in the firm’s effectiveness in contesting the campaign and consequently in the firm’s CSR investments depending on the relative values of *B* and *L*. For relatively low (high) *B* values the optimal effort of the activist and the firm are decreasing (increasing) in the firm’s CSR investments. Relatively low benefits for the activist when the campaign succeeds, followed by an increase in CSR, which causes the firm’s effectiveness in contesting the campaign to increase, reduce the activist’s incentive to exert greater effort and, through this, they reduce the firm’s incentive to exert greater effort to contest the campaign (see SA.1-SA.3 in S1 Appendix for proofs).

Substituting the optimal level of effort of firm *H* and the activist (Eqs (12) and (13)) into Eq (8) we get:

$$\theta =\frac{B-L}{(B-L)+\alpha (Z)\left(\frac{4}{9}\right)}$$ (14)

The probability of campaign success increases in the benefit the activist can realize when the campaign succeeds, *B*, and decreases in both the benefit the activist can realize when the campaign fails, *L*, and in the firm’s effectiveness in contesting the campaign, *a*(*Z*).

### Stage 2—The activist decides whether to monitor the firm

When firm *H* claims to provide the credence quality attribute, *s*_*c*_ = 1, the activist decides whether to monitor the firm or not. If the activist chooses to monitor the firm, she will find the actual quality produced by the firm, *s*_*a*_, and, by comparing it to the quality claimed, *s*_*c*_, she can determine whether fraudulent quality claims have been made. If fraudulent quality claims were made (i.e., *s*_*c*_ > *s*_*a*_ = 0), she will launch a campaign against firm *H* to exert social pressure to remove the erroneous quality claims. If she chooses not to monitor the firm, she will not know whether fraudulent quality claims have been made and realizes no benefit. The choice of whether to monitor the firm is made by comparing the payoffs realized when she monitors firm *H*, *E* (Π_*A*_)^*M*^, to the payoff realized when she does not monitor the firm, $\Pi_{A}^{NM}$. That is, if $E{\left(\Pi_{A}\right)}^{M}=\mu \left(E{\left(\Pi_{A}\right)}^{MC}\right)+\left(1-\mu \right)\Pi_{Aa}^{M}>\;\Pi_{A}^{NM}$, the activist will choose to monitor the firm, while if $E{\left(\Pi_{A}\right)}^{M}\leq \;\Pi_{A}^{NM}$, the activist will choose not to monitor the firm. Recall that the parameter *μ* denotes the likelihood that firm *H* has made false quality claims. The activist will, thus, monitor the firm when:

$$\begin{array}{l} E{\left(\Pi_{A}\right)}^{M}=\mu \left[\theta \text{B}+\left(1-\theta \right)L-e\right]+\left(1-\mu \right)\beta -c_{m}>\;\Pi_{A}^{NM}=0\Rightarrow \\ \Rightarrow \mu \left[\left(\frac{\left(B-L\right)}{\left(B-L\right)+\alpha \left(Z\right)\left(\frac{4}{9}\right)}\right)B+\left(\frac{\alpha \left(Z\right)\left(\frac{4}{9}\right)}{\left(B-L\right)+\alpha \left(Z\right)\left(\frac{4}{9}\right)}\right)L-\frac{{\left(\left(B-L\right)\right)}^{2}\alpha \left(Z\right)\left(\frac{4}{9}\right)}{{\left[\left(B-L\right)+\alpha \left(Z\right)\left(\frac{4}{9}\right)\right]}^{2}}\right]\\ +\left(1-\mu \right)\beta -c_{m}>0\Rightarrow \alpha \left(Z\right)<\alpha_{c}^{A} \end{array}$$ (15)

where, $\alpha_{c}^{A}=\frac{9}{4}(-B+L+\frac{{\left(B-L\right)}^{3}\mu }{\sqrt{{\left(B-L\right)}^{3}\mu (c_{m}+\beta (-1+\mu )-L\mu )}})$ is the critical value that makes the activist indifferent between monitoring and not monitoring the firm; thus, as long as $\alpha \left(Z\right)<\alpha_{c}^{A}$ the activist will monitor the firm.

**Fig 2:**
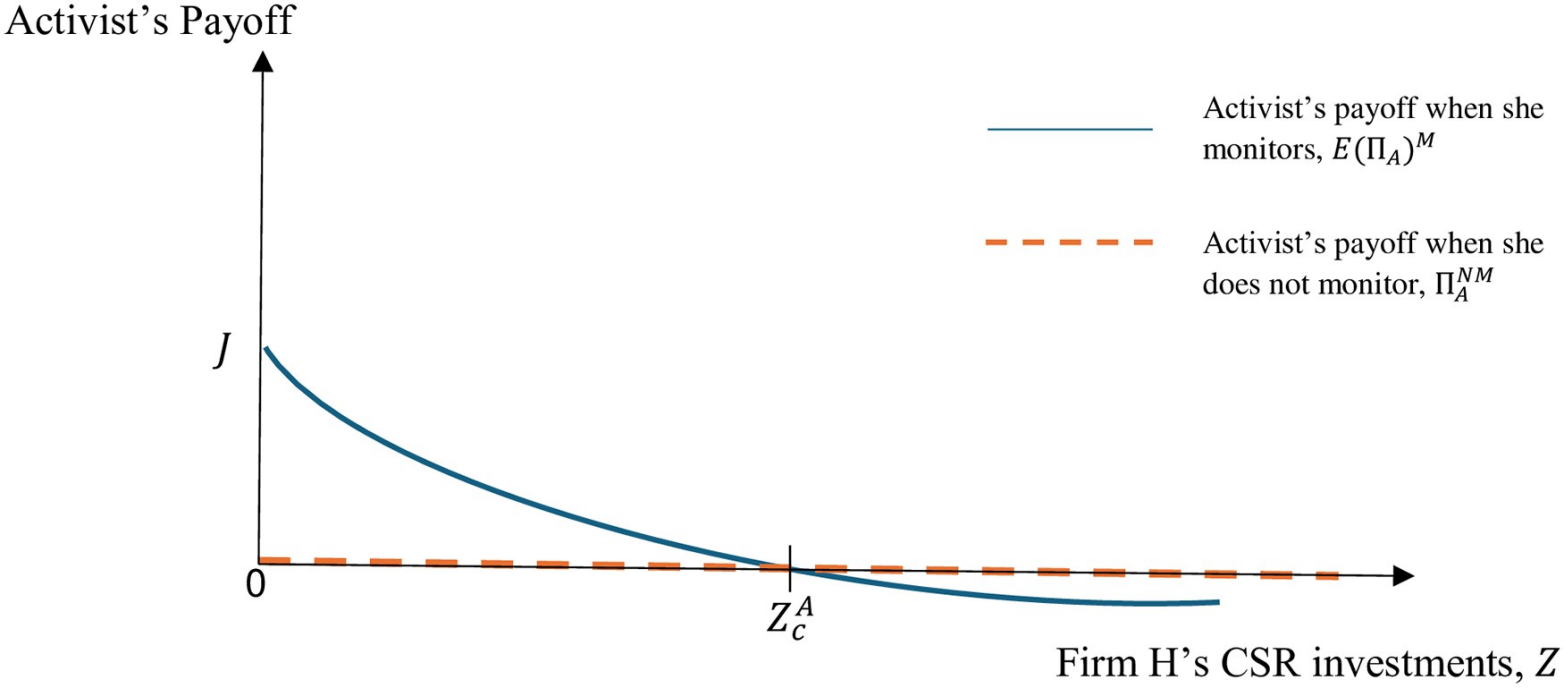
The activist’s payoffs when she monitors and does not monitor firm *H* for different *Z* values when *α*(*Z*) = *δ* + *Z*.

Recall that, the greater are the firm’s CSR investments, the greater is the effectiveness of its effort in contesting the campaign, *α*(*Z*), which, in turn, decreases the probability of campaign success, making it less likely that the activist will monitor the firm. The level of CSR investments *Z* that makes the activist indifferent between monitoring and not monitoring the firm, depends on the functional form of *α*(*Z*). For instance, for the simple functional form *α*(*Z*) = *δ* + *Z*, where *δ* ≥ 1, the value of *Z* that makes the activist indifferent between monitoring and not monitoring the firm is given by $Z_{c}^{A}=\frac{1}{4}(-9B-4\delta +9L+\frac{9{\left(B-L\right)}^{3}\mu }{\sqrt{{\left(B-L\right)}^{3}\mu (c_{m}-L\mu +(-1+\mu )\beta )}})$. Fig 2 depicts the activist’s payoffs under monitoring and no monitoring for different values of firm *H*’s CSR investments, *Z*, when *α*(*Z*) = *δ* + *Z* and where $J=\frac{(81B^{3}-162B^{2}L+8\delta (2\delta -9L)L+9BL(8\delta +9L))\mu }{{\left(9B+4\delta -9L\right)}^{2}}-c_{m}+\beta -\mu \beta$.

Even though we do not explicitly model firm *H*’s CSR investment decision (recall that in our model the firm’s CSR investments are sunk when the firm makes its production decisions), it is easy to see in Fig 2 that the firm could use its CSR investments to induce the desired behavior by the activist, i.e., to be monitored or not be monitored by the activist. Thus, by choosing a level of CSR such that $Z\geq Z_{c}^{A}$, the firm can make it unprofitable for the activist to monitor the firm.

Whether this would be an optimal strategy for the firm would depend, among other things, on the cost of CSR and the effect it has on the firm’s effort in contesting the activist’s campaign. The more costly is CSR and the weaker its impact on the firm’s effectiveness in contesting attacks against it, the less likely it becomes that the firm will be able to use its CSR investments to avoid being monitored by the activist.

### Stage 1—Firm *H* chooses its product quality and makes quality claims

#### Firm *H* makes quality claims

In stage one, firm *H* chooses either to claim a quality for good *h* that is greater than its actual quality, *s*_*c*_ > *s*_*a*_ (which implies that it does not provide the credence attribute but claims to do so, i.e., *s*_*a*_ = 0 and *s*_*c*_ = 1), or to claim its actual quality, *s*_*c*_ = *s*_*a*_ (which implies that either *s*_*c*_ = *s*_*a*_ = 0 or *s*_*c*_ = *s*_*a*_ = 1), by comparing the profits associated with each choice. Firm *H* chooses the quality claims it will make by comparing the profits realized when it mispresents its quality, *E*(Π_*h*_), with the profits realized when it truthfully claims its quality, Π_*ha*_. Solving the game backwards and using the solutions from stage two, firm *H*’s decision whether to misrepresent its quality is examined under two cases, namely, when the activist will monitor the firm (Scenario A) and when the activist will not monitor the firm (Scenario B).

#### Scenario A: The activist monitors the firm $\left(\alpha \left(Z\right)<\alpha_{c}^{A}\right)$

Under this scenario, firm *H* chooses the quality claims it will make by comparing the profits when it misrepresents its product quality (and the activist launches a campaign against firm *H*) *E*(Π_*h*_)^*MC*^ = *θ*(*π*_*hs*_) + (1 − *θ*)(*π*_*hf*_) − *f* with the profits realized when firm *H* truthfully claims its quality, *Π*_*ha*_ = *π*_*ha*_ − *F*_*h*_, where, as noted earlier, *F*_*h*_ are the fixed costs incurred for the provision of the credence attribute. When *E*(Π_*h*_)^*MC*^ > *Π*_*ha*_, firm *H* chooses *s*_*c*_ > *s*_*a*_, while when ${E(\Pi_{h})}^{MC}\leq \Pi_{ha}$, it chooses *s*_*c*_ = *s*_*a*_. In particular, firm *H* chooses *s*_*c*_ > *s*_*a*_ if:

$$\begin{array}{l} E{\left(\Pi_{h}\right)}^{MC}>\Pi_{ha}\Rightarrow \theta \left(\pi_{hs}\right)+\left(1-\theta \right)\left(\pi_{hf}\right)-f\geq \pi_{ha}-F_{h}\Rightarrow \\ \left(\frac{\left(B-L\right)}{\left(B-L\right)+\alpha \left(Z\right)\left(\frac{4}{9}\right)}\right)\;0+\;\left(\frac{\alpha \left(Z\right)\left(\frac{4}{9}\right)}{\left(B-L\right)+\alpha \left(Z\right)\left(\frac{4}{9}\right)}\right)\left(\frac{4}{9}\right)-\frac{\left(B-L\right)\alpha \left(Z\right){\left(\frac{4}{9}\right)}^{2}}{{\left[\left(B-L\right)+\alpha \left(Z\right)\left(\frac{4}{9}\right)\right]}^{2}}\\ >\left(\frac{4}{9}\right)-F_{h}\Rightarrow \alpha \left(Z\right)>\alpha_{c}^{H} \end{array}$$ (16)

where $\alpha_{c}^{H}=\frac{4B-9BF_{h}-4L+9F_{h}L+2\sqrt{4B^{2}-9B^{2}F_{h}-8BL+18BF_{h}L+4L^{2}-9F_{h}L^{2}}}{4F_{h}}$ is the critical value that makes the firm indifferent between misrepresenting and truthfully claiming its quality when it is monitored by the activist. When firm *H* expects it will be monitored by the activist, it will find it optimal to misrepresent its quality when $\alpha \left(Z\right)>\alpha_{c}^{H}$; that is, the greater is the effectiveness of the firm in contesting the campaign, the more likely it is that it will misrepresent its quality. The likelihood that firm *H* will find it optimal to misrepresent its product quality also increases in the cost of providing the credence attribute, *F*_*h*_, that is, $\frac{\partial \alpha_{c}^{H}}{\partial F_{h}}<0$ (see SA.4. in S1 Appendix for a proof). Please note that this result holds even when CSR has no effect on the firm’s effort to contest the activist’s campaign, that is, when *a*(*Z*) = 1. In this case, substituting for *a*(*Z*) = 1 in Eq (16) the inequality becomes ${E(\Pi_{h})}^{MC}>\Pi_{ha}\Rightarrow \left(\frac{\left(\frac{4}{9}\right)}{\left(B-L\right)+\frac{4}{9}}\right)\frac{4}{9}-\frac{\left(B-L\right)*{\left(\frac{4}{9}\right)}^{2}}{{\left(\left(B-L\right)+\frac{4}{9}\right)}^{2}}>\frac{4}{9}-F_{h}$, which shows that the greater (lower) is the cost of providing the credence attribute *F*_*h*_, the more (less) likely it becomes that the inequality will hold, i.e., that committing fraud will be more (less) profitable for the firm than truthfully claiming its quality.

**Fig 3:**
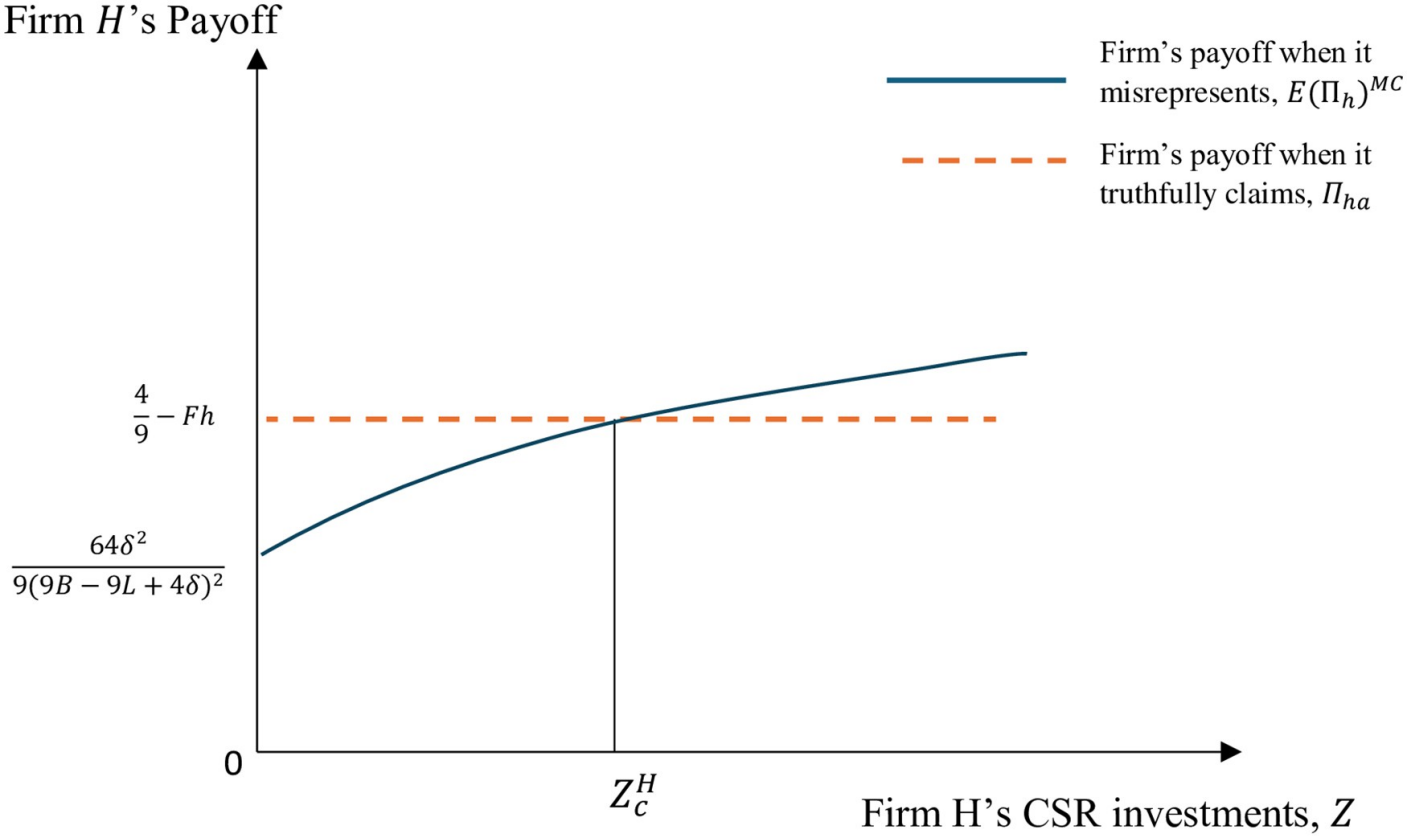
Firm *H*’s payoffs when it misrepresents and when it truthfully claims its product quality for different CSR investment values when *α*(*Z*) = *δ* + *Z*.

As noted earlier, the greater are the firm’s CSR investments, the more effective is the firm in contesting the campaign. As a result, the probability of campaign success decreases in the firm’s CSR investments, making it more likely that the firm will find it optimal to mispresent its quality. The level of CSR investments that make firm *H* indifferent between misrepresenting and truthfully claiming its product quality depends on the functional form of *α*(*Z*). For instance, when *α*(*Z*) = *δ* + *Z*, where *δ* ≥ 1, $Z_{c}^{H}=\frac{4B-9BF_{h}-4{\delta F}_{h}+2\sqrt{-(-4+9F_{h}){\left(B-L\right)}^{2}}-4L+9F_{h}L}{4F_{h}}$. This case is depicted in Fig 3 which shows firm *H*’s payoffs when it misrepresents and when it truthfully claims its product quality for different values of the firm’s CSR investments, *Z*.

#### Scenario B: The activist does not monitor the firm ($\alpha \left(Z\right)>\alpha_{c}^{A}$)

When the activist does not monitor the firm, firm *H* will always find it optimal to misrepresent its quality given that the public is unable to observe that the firm has engaged in fraudulent behavior due to the credence nature of its quality attribute. This is because, in this case, the profits the firm realizes when it misrepresent its product quality are strictly greater than the profits the firm realizes when it truthfully claims its product quality, that is, $\Pi_{hc}^{NM}=\pi_{hc}>\Pi_{ha}=\pi_{ha}-F_{h}\Rightarrow \frac{4}{9}>\frac{4}{9}-F_{h}$ for any *F*_*h*_ > 0.

**Fig 4:**
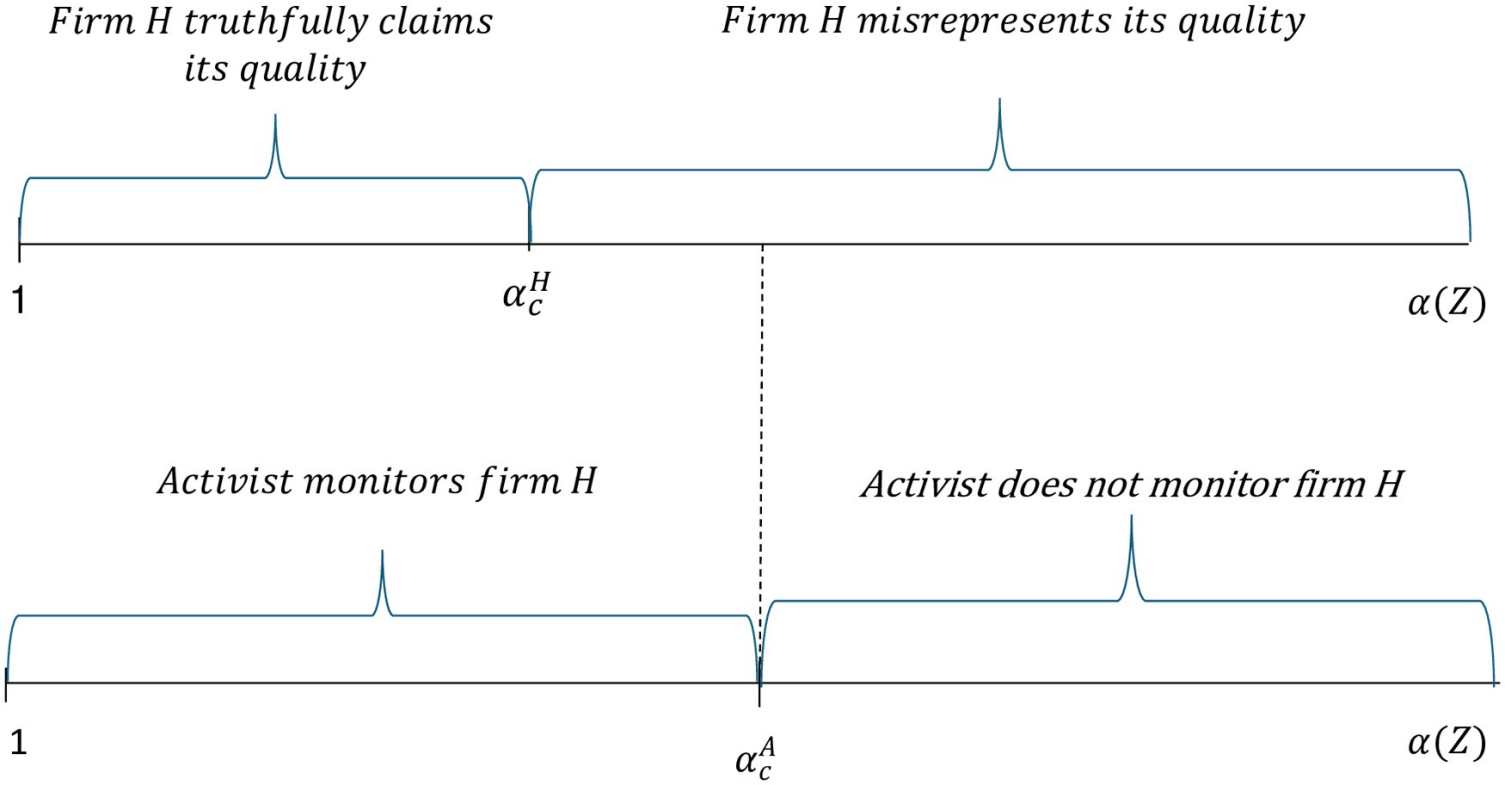
Firm *H*’s decision to misrepresent its product quality and the activist’s decision to monitor firm *H* for different *α*(*Z*) values.

**Fig 5:**
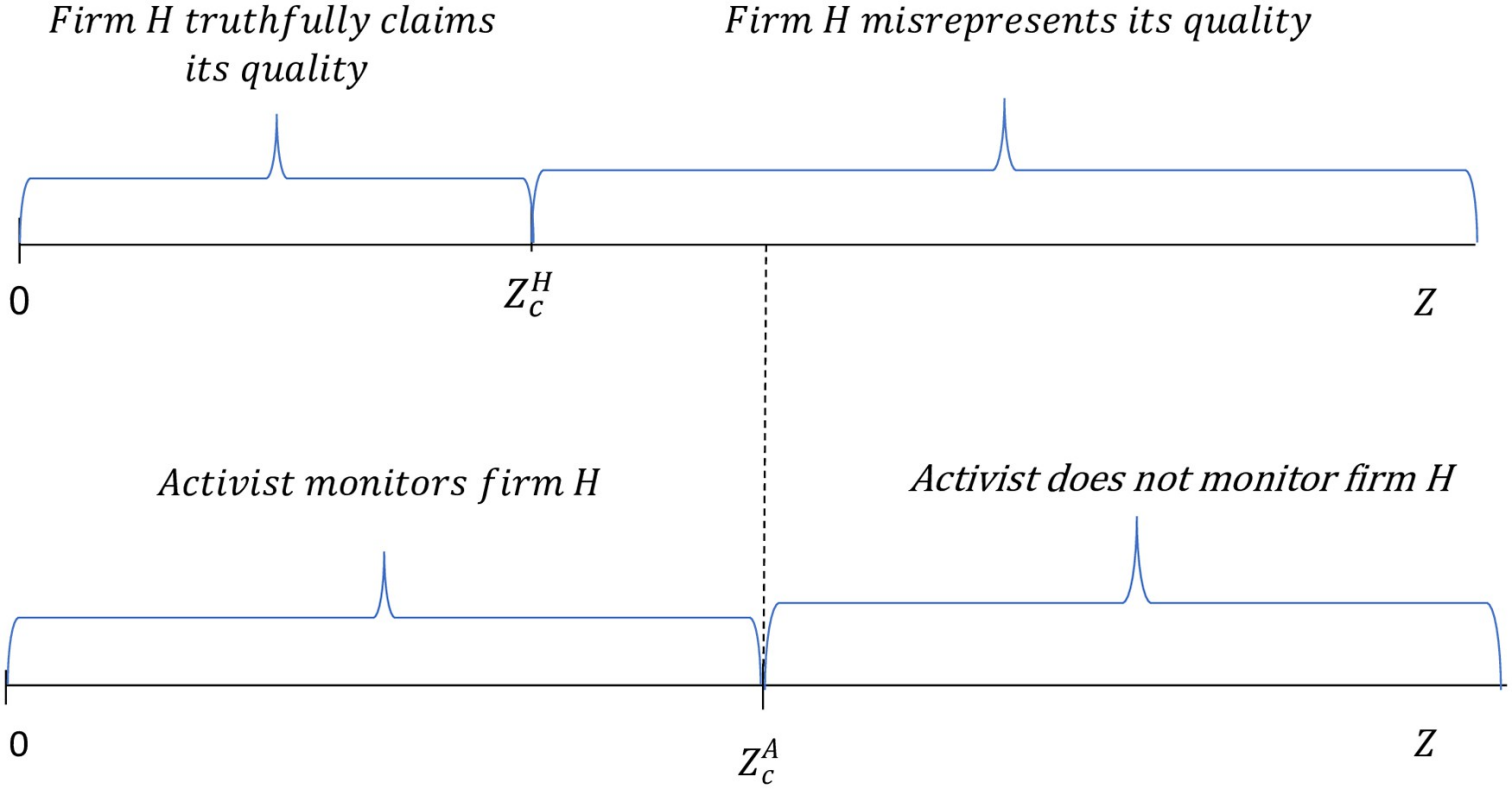
Firm *H*’s decision to misrepresent its product quality and the activist’s decision to monitor firm *H* for different *Z* values when *α*(*Z*) = *δ* + *Z*.

Fig 4 summarizes the activist’s and firm *H*’s decisions under scenarios A and B for different values of firm *H*’s effectiveness in contesting the campaign *α*(*Z*), while Fig 5 summarizes these decisions for different CSR values when firm *H*’s effectiveness in contesting the campaign is given by *α*(*Z*) = *δ* + *Z*.

As can be seen in Fig 4, if firm *H*’s effectiveness in contesting the campaign is above the threshold value $\alpha_{c}^{A}$, the activist will find it optimal to not monitor the firm and firm *H* will find it optimal to misrepresent its quality. If firm *H*’s effectiveness in contesting the campaign is below the threshold value $\alpha_{c}^{A}$, the activist will find it optimal to monitor the firm and firm *H* will either truthfully claim its quality or misrepresent it depending on whether its effectiveness in contesting the campaign is below or above the threshold value $\alpha_{c}^{H}$, respectively.

A simple numerical example where parameter values satisfy the model restrictions shows the existence of threshold values that satisfy the condition $1{<\alpha }_{c}^{H}<\alpha_{c}^{A}$ and give rise to the outcomes depicted in Fig 4. Thus, when *B* = 2, *β* = 1.5, *L* = 1, *F*_*h*_ = 0.12, *μ* = 0.5, and *c*_*m*_ = 1.258, the threshold value that makes the activist indifferent between monitoring and not monitoring firm *H* is $\alpha_{c}^{A}=15.54$ while the threshold value that makes firm *H* indifferent between truthfully claiming and misrepresenting its quality is equal to $\alpha_{c}^{H}=13.2$. It is easy to confirm that at the above threshold values Eqs (15) and (16) are met as equalities, i.e., $E{\left(\Pi_{A}\right)}^{M}=\Pi_{A}^{NM}=0$ and *E*(Π_*h*_)^*MC*^ = *Π*_*ha*_ = 0.324, respectively. Recall that when the activist does not monitor the firm, she earns zero profits while the maximum profit firm *H* can earn is when it misrepresents its quality and is not monitored by the activist, $\Pi_{hc}^{NM}=\frac{4}{9}$.

As shown in Fig 5, the activist’s and firm *H*’s decisions can also be depicted in terms of the firm’s CSR investments, *Z*, if we assume a specific relationship between these investments and the firm’s effectiveness in contesting the campaign. When firm *H*’s CSR effectiveness in contesting the campaign is given by the simple functional form *α*(*Z*) = *δ* + *Z* we can show the existence of critical threshold values that satisfy the condition $0<Z_{c}^{H}<Z_{c}^{A}$ and give rise to the outcomes depicted in Fig 5. Thus, for the parameter values considered above, and for *δ* = 1.5, the threshold value of CSR investments that makes the activist indifferent between monitoring and not monitoring the firm is equal to $Z_{c}^{A}=14.04$ while the threshold value that makes firm *H* indifferent between truthfully claiming and misrepresenting its quality is equal to $Z_{c}^{H}=11.7$.

#### Firm *H* chooses its product quality

Firm *H* chooses the quality of good *h* it will produce to maximize its profits. Using the equilibrium conditions derived earlier, the choice of optimal product quality depends on whether firm *H* will choose to misrepresent its quality, $s_{c}^{*}>s_{a}$ (Case I), or it will choose to claim its actual quality, $s_{c}^{*}=s_{a}$ (Case II).

Case I: Firm *H* chooses $s_{c}^{*}>s_{a}\;(\alpha \left(Z\right)>\alpha_{c}^{H})$

When firm *H* finds it optimal to misrepresent its product quality, that is when $s_{c}^{*}>s_{a}$, it does not provide the credence attribute, *s*_*a*_ = 0. Recall that, if the firm provided the credence attribute there would be no misrepresentation of its true quality as $s_{c}^{*}=s_{a}=1$.

Case II: Firm *H* chooses $s_{c}^{*}=s_{a}\;(\alpha \left(Z\right)\leq \alpha_{c}^{H})$

When firm *H* finds it optimal to truthfully claim its quality, it can either choose not to provide the credence attribute, *s*_*a*_ = 0, or to provide the credence attribute, *s*_*a*_ = 1, by comparing the profits associated with each choice. If the firm does not provide the credence attribute, it realizes zero profits as discussed previously, given that it competes in prices with firm *K*. It is straightforward to show that the decision to provide the credence attribute depends on the level of fixed costs associated with its provision. In particular, firm *H* will find it optimal to provide the credence attribute when $\pi_{ha}-F_{h}=\frac{4}{9}-F_{h}>0$, which holds true when fixed costs are $F_{h}<\frac{4}{9}$, and will not provide the credence attribute when $F_{h}\geq \frac{4}{9}$. Thus, the lower are the costs associated with the provision of the credence attribute, the more likely it is that the firm will find it optimal to provide the credence attribute and truthfully claim its product quality.

## 4. Model extensions and conclusions

The study developed an analytical model to examine the relationship between a firm’s CSR investments, its decision to misrepresent its product quality, and an activist’s decision to target the firm to uncover and remove fraudulent quality claims. Analytical results show that the greater are the firm’s CSR investments, the less likely it is that the activist will find it optimal to target the firm, and the more likely it is that the firm will misrepresent its product quality. The results also show that even when the activist finds it optimal to monitor the firm, the firm may still find it optimal to misrepresent its product quality. This outcome is more likely to emerge when the firm’s CSR investments are relatively large, making the firm more effective in contesting a campaign against it, and increasing the probability of campaign failure for the activist. The results also show that the greater is the cost of providing the credence attribute, the more likely it is that the firm will find it optimal to misrepresent its product quality (and not actually provide the credence attribute). Moreover, although the study does not explicitly model the firm’s CSR investment decision, the analysis shows that the firm could strategically use its CSR investments to affect the activist’s decision to monitor the firm. That is, increasing its CSR investments just above a critical threshold value can induce the desired behavior by the activist, i.e., to not monitor the firm.

The analysis also sheds some light on the incentives of rival firms competing with the firm that considers providing the credence quality attribute. In the vertically differentiated market considered in our analysis, when the firm that provides the credence good makes false quality claims, the profits of its rival rise due to increased product differentiation in the market. Thus, in this type of market, rival firms do not have an incentive to expose fraudulent quality claims and/or collaborate with an activist whose objective is to expose firms that make fraudulent quality claims.

It is important to mention that while our study focuses on fraud in the food sector, our analysis and results are more general and could apply to other sectors that provide credence attributes that share similar characteristics with the food products considered in this study; that is goods whose credence attribute is the differentiating quality attribute in the relevant vertically differentiated markets. As we discuss in the introduction, our focus on the food sector is motivated by the proliferation of food fraud incidents and the recent surge in activism, including legal activism against fraudulent and misleading food quality claims, in the food sector.

Our results are based on a number of assumptions. First, we have assumed that as long as there is no challenge to firm *H*’s claims, consumers believe that the firm provides the credence attribute when it claims to do so. In the vertically differentiated market that we model, if consumers do not believe firm *H*’s claims and assume that it does not provide the credence attribute, they won’t pay a premium for *H*’s product (the quality parameter *s* in Eq (1) will be equal to zero). In this case, when the firm indeed tries to mislead the public, the presence of the activist will not change market outcomes. If, however, the firm does provide the credence attribute, but consumers do not believe the firm’s claims, the activist could play an important role in informing the public about the true nature of the firm’s product.

Second, we have assumed that when the activist finds it optimal to monitor the firm, she can accurately detect fraud. Relaxing this assumption and assuming instead that the activist cannot accurately detect fraud, changes the expected cost of making false quality claims and thus, changes firm *H*’s incentive to make false quality claims about its product quality. If the activist cannot accurately evaluate the firm’s actual quality, she may launch a campaign against the firm even when the firm has not misrepresented its quality or not take any action when the firm has misrepresented its quality. If the firm anticipates that the former (latter) outcome is more likely, it will be less (more) likely to misrepresent its quality. Third, we have assumed that the uncovering of fraudulent claims by the activist does not lead to other penalties being incurred by the firm. Obviously, if in addition to consumer backlash the firm faces penalties from regulators, it is less likely that it will find it optimal to misrepresent its product quality. Finally, our analysis was conducted under the assumption that when the activist launches a successful campaign, the firm can no longer make fraudulent claims and provides the basic product. If, instead, as a result of a successful campaign against it, the firm starts providing the credence attribute, one has to consider under what conditions the public will believe the firm–consumers may be uncertain in this case, assigning a positive likelihood that the firm’s product is low rather than high quality.

Several research extensions of the study are important to highlight. We examined the case where the firm’s quality choice is binary; it either provides or does not provide the quality attribute as is the case when the credence quality attribute is linked to the firm’s production process (e.g., organic, sustainable, local, fair trade). It would be interesting to examine how the firm’s CSR investments and the presence of the activist impact the quality level of the credence good supplied in the market when the firm’s quality choice is continuous, which is the case when a firm decides on the nutritional content of its food or the level of its fortification. It would also be interesting to examine the effectiveness of activists who might have objectives unobserved by other market players when choosing their targets (e.g., the activist may oppose large corporations) rather than to uncover and remove false quality claims about the product quality. This uncertainty about the true objectives of the activist might impact the activist’s ability to launch successful campaigns and, in turn, firm incentives to make false quality claims.

Finally, it would be interesting to explicitly model the firm’s CSR investment decision under different scenarios. For instance, our results show that the firm could strategically choose the level of its CSR investments to influence the behavior of the activist and increase its profits. However, CSR investments are costly for the firm and provide an advantage when the public perceives them as benefiting society rather than providing a strategic advantage to the firm. An activist could take action to undermine the impact of the firm’s CSR campaigns, which, in turn, would impact the firm’s incentive to invest in CSR. These research extensions are the focus of future research.

## Supporting information

S1 Appendix (DOCX)
