# Supplementary material for: The economic impacts of social activism and corporate social responsibility on food fraud
Source: PLoS One. 2024 Jun 11;19(6):e0304153. doi: 10.1371/journal.pone.0304153 (PMC11166316; doi:10.1371/journal.pone.0304153)
Supplement: S1 Appendix — (DOCX) [file pone.0304153.s001.docx]

**S1 Appendix**

**SA.1. The effect of B and L on the optimal effort of the activist,** $\boldsymbol{e}^{\boldsymbol{*}}$

$\frac{\partial e^{*}}{\partial B}=\frac{288{a(Z)}^{2} \left( B-L \right)}{\left( 4\alpha\left( Z \right)+9B-9L \right)^{3}}$>0 and $\frac{\partial e^{*}}{\partial L}=-\frac{288{a\left( Z \right)}^{2} \left( B-L \right)}{\left( 4\alpha\left( Z \right)+9B-9L \right)^{3}}<0$.

**SA.2. The effect of B and L on the optimal effort of the firm,** $\boldsymbol{f}^{\boldsymbol{*}}$

$\frac{\partial f^{*}}{\partial B}=\frac{16a\left( Z \right)\left( 4\alpha\left( Z \right)-9B+9L \right)}{\left( 4\alpha\left( Z \right)+9B-9L \right)^{3}}$ and $\frac{\partial f^{*}}{\partial L}=-\frac{16a\left( Z \right)\left( 4\alpha\left( Z \right)-9B+9L \right)}{\left( 4\alpha\left( Z \right)+9B-9L \right)^{3}}$

When $1<a(Z)\leq\frac{9(B-L)}{4}$, $\frac{\partial f^{*}}{\partial B}=\frac{16a\left( Z \right)\left( 4\alpha\left( Z \right)-9B+9L \right)}{\left( 4\alpha\left( Z \right)+9B-9L \right)^{3}}\leq0$ and $\frac{\partial f^{*}}{\partial L}=-\frac{16a\left( Z \right)\left( 4\alpha\left( Z \right)-9B+9L \right)}{\left( 4\alpha\left( Z \right)+9B-9L \right)^{3}}\geq0$ while when $a\left( Z \right)>\frac{9(B-L)}{4}$ , $\frac{\partial f^{*}}{\partial B}=\frac{16a\left( Z \right)\left( 4\alpha\left( Z \right)-9B+9L \right)}{\left( 4\alpha\left( Z \right)+9B-9L \right)^{3}}>0$ and $\frac{\partial f^{*}}{\partial L}=-\frac{16a\left( Z \right)\left( 4\alpha\left( Z \right)-9B+9L \right)}{\left( 4\alpha\left( Z \right)+9B-9L \right)^{3}}<0$.

**SA.3. The effect of firm** $H$**’s effectiveness in contesting the campaign and its CSR investments on the optimal effort of the activist,** $\boldsymbol{e}^{\boldsymbol{*}}$**, and the firm,** $\boldsymbol{f}^{\boldsymbol{*}}$

When $L<B\leq\frac{1}{9}(4 a(Z)+9L)$, $\frac{\partial e^{*}}{\partial\alpha(Z)}=-\frac{36\left( B-L \right)^{2}\left( 4\alpha\left( Z \right)-9B+9L \right)}{\left( 4\alpha\left( Z \right)+9B-9L \right)^{3}}\leq0$ and $\frac{\partial f^{*}}{\partial\alpha(Z)}=-\frac{16\left( B-L \right)\left( 4\alpha\left( Z \right)-9B+9L \right)}{\left( 4\alpha\left( Z \right)+9B-9L \right)^{3}}\leq0$. Since the firm’s effectiveness in contesting the campaign increases in the level of CSR investments, $\alpha^{'}\left( Z \right)>0$, it follows that the optimal efforts of the activist, $e^{*}$, and the firm, $f^{*}$, are decreasing in CSR, $Z$.

When $B>\frac{1}{9}(4 a(Z)+9L)$, $\frac{\partial e^{*}}{\partial\alpha(Z)}=-\frac{36\left( B-L \right)^{2}\left( 4\alpha\left( Z \right)-9B+9L \right)}{\left( 4\alpha\left( Z \right)+9B-9L \right)^{3}}>0$ and $\frac{\partial f^{*}}{\partial\alpha(Z)}=-\frac{16\left( B-L \right)\left( 4\alpha\left( Z \right)-9B+9L \right)}{\left( 4\alpha\left( Z \right)+9B-9L \right)^{3}}>0$ and because $\alpha^{'}\left( Z \right)>0$, the optimal efforts of the activist, $e^{*}$, and the firm, $f^{*}$, are increasing in CSR, $Z$.

Similarly, when $1<a(Z)\leq\frac{9(B-L)}{4}$ , $\frac{\partial e^{*}}{\partial\alpha(Z)}=-\frac{36\left( B-L \right)^{2}\left( 4\alpha\left( Z \right)-9B+9L \right)}{\left( 4\alpha\left( Z \right)+9B-9L \right)^{3}}\geq0$ and $\frac{\partial f^{*}}{\partial\alpha(Z)}=-\frac{16\left( B-L \right)\left( 4\alpha\left( Z \right)-9B+9L \right)}{\left( 4\alpha\left( Z \right)+9B-9L \right)^{3}}\geq0.$ Since the firm’s effectiveness in contesting the campaign increases in the level of CSR investments, $\alpha^{'}\left( Z \right)>0$, it follows that the optimal efforts of the activist, $e^{*}$, and the firm, $f^{*}$, are increasing in CSR, $Z$.

When $a\left( Z \right)>\frac{9(B-L)}{4}$, $\frac{\partial e^{*}}{\partial\alpha(Z)}=-\frac{36\left( B-L \right)^{2}\left( 4\alpha\left( Z \right)-9B+9L \right)}{\left( 4\alpha\left( Z \right)+9B-9L \right)^{3}}<0$ and $\frac{\partial f^{*}}{\partial\alpha(Z)}=-\frac{16\left( B-L \right)\left( 4\alpha\left( Z \right)-9B+9L \right)}{\left( 4\alpha\left( Z \right)+9B-9L \right)^{3}}<0$. Since $\alpha^{'}\left( Z \right)>0$, it follows that the optimal efforts of the activist, $e^{*}$, and the firm, $f^{*}$, are decreasing in CSR, $Z$.

**SA.4. The effect of the cost of providing the credence attribute,** $\boldsymbol{F}_{\boldsymbol{h}}$**, on the critical value of the effectiveness of the effort of the firm,** $\boldsymbol{\alpha}_{\boldsymbol{c}}^{\boldsymbol{H}}$

$\frac{\partial\alpha_{c}^{H}}{\partial F_{h}}=-\frac{\left( B-L \right)\left( B\left( 8-9F_{h} \right)+8\sqrt{-\left( -4+9F_{h} \right)\left( B-L \right)^{2}}+\left( -8+9F_{h} \right)L \right)}{8{F_{h}}^{2}\sqrt{-\left( -4+9F_{h} \right)\left( B-L \right)^{2}}}<0$.

**S2 Numerical Example**

For the simple numerical example used in our paper the following parameter values were used that satisfy all model restrictions: $B=2$, $\beta=1.5$, $L=1$, $F_{h}=0.12$, $\mu=0.5$, and $c_{m}=1.258$. Substituting these values into the expressions for the threshold values $\alpha_{c}^{A}$ and $\alpha_{c}^{H}$ yields:

$$\alpha_{c}^{A}=\frac{9}{4}\left( -B+L+\frac{\left( B-L \right)^{3}\mu}{\sqrt{\left( B-L \right)^{3}\mu\left( c_{m}+\beta\left( -1+\mu\right)-L\mu\right)}} \right)=15.54$$

$$\alpha_{c}^{H}=\frac{4B-9BF_{h}-4L+9F_{h}L+2\sqrt{4B^{2}-9B^{2}F_{h}-8BL+18BF_{h}L+4L^{2}-9F_{h}L^{2}}}{4F_{h}}=13.2$$

The above values satisfy the condition $1{<\alpha}_{c}^{H}<\alpha_{c}^{A}$ and give rise to the outcomes depicted in figure 4. Since $\alpha_{c}^{A}$ is the threshold value that makes the activist indifferent between monitoring and not monitoring firm $H$ while $\alpha_{c}^{H}$ is the threshold value that makes firm $H$ indifferent between truthfully claiming and misrepresenting its quality, it is straightforward to show that for $\alpha_{c}^{A}=15.54$ and $\alpha_{c}^{H}=13.2$ equations (15) and (16) are met as equalities, i.e., $E{(\Pi_{A})}^{M}= \Pi_{A}^{NM}=0$ and ${E(\Pi_{h})}^{MC}=\Pi_{ha}=0.324$, respectively.

When firm $H$’s CSR effectiveness in contesting the campaign is given by the simple functional form $\alpha\left( Z \right)=\delta+Z$ we can show the existence of critical threshold values that satisfy the condition $0<Z_{c}^{H}<Z_{c}^{A}$ and give rise to the outcomes depicted in figure 5. Thus, for the parameter values considered above, and for $\delta=1.5$, the threshold value of CSR investments that makes the activist indifferent between monitoring and not monitoring the firm is equal to

$Z_{c}^{A}=\frac{1}{4}(-9B-4\delta+9L+\frac{9{(B-L)}^{3}\mu}{\sqrt{{(B-L)}^{3}\mu(c_{m}-L\mu+(-1+\mu)\beta)}})=14.04$ while the threshold value that makes firm $H$ indifferent between truthfully claiming and misrepresenting its quality is equal to $Z_{c}^{H}=\frac{4B-9BF_{h}-4{\delta F}_{h}+2\sqrt{-(-4+9F_{h}){(B-L)}^{2}}-4L+9F_{h}L}{4F_{h}}=11.7$.
